# Retinoic acid prevents immunogenicity of milk lipocalin Bos d 5 through binding to its immunodominant T-cell epitope

**DOI:** 10.1038/s41598-018-19883-0

**Published:** 2018-01-25

**Authors:** Karin Hufnagl, Debajyoti Ghosh, Stefanie Wagner, Alessandro Fiocchi, Lamia Dahdah, Rodolfo Bianchini, Nina Braun, Ralf Steinborn, Martin Hofer, Marion Blaschitz, Georg A. Roth, Gerlinde Hofstetter, Franziska Roth-Walter, Luis F. Pacios, Erika Jensen-Jarolim

**Affiliations:** 1 The Interuniversity Messerli Research Institute of the University of Veterinary Medicine Vienna, Medical University Vienna and University Vienna, Vienna, Austria; 2 University of Cincinnati College of Medicine, Ohio, USA; 3 Childrens Hospital Bambino Gesù, Rome, Italy; 4 Genomics Core Facility, VetCore, University of Veterinary Medicine, Vienna, Austria; 5 Center for Anthropogenic Infections, Division for Public Health, Austrian Agency for Health and Food Safety, Vienna, Austria; 6 Department of Anesthesiology, General Intensive Care and Pain Medicine, Medical University of Vienna, Vienna, Austria; RAIC Laboratory 13C1, Medical University of Vienna, Vienna, Austria; 7 Department of Natural Systems and Resources, ETSI Montes, Technical University of Madrid, and Centro de Biotecnología y Genómica de Plantas (CBGP) UPM-INIA, Campus de Montegancedo UPM, Madrid, Spain; 8 Institute of Pathophysiology and Allergy Research, Center for Pathophysiology, Infectiology and Immunology, Medical University Vienna, Vienna, Austria

## Abstract

The major cow’s milk allergen Bos d 5 belongs to the lipocalin protein family, with an intramolecular pocket for hydrophobic ligands. We investigated whether Bos d 5 when loaded with the active vitamin A metabolite retinoic acid (RA), would elicit differential immune responses compared to the unloaded state. By *in silico* docking an affinity energy of −7.8 kcal/mol was calculated for RA into Bos d 5. Loading of RA to Bos d 5 could be achieved *in vitro*, as demonstrated by ANS displacement assay, but had no effect on serum IgE binding in tolerant or challenge-positive milk allergic children. Bioinformatic analysis revealed that RA binds to the immunodominant T-cell epitope region of Bos d 5. In accordance, Bos d 5 significantly suppressed the CD3+ CD4+ cell numbers, proliferative response and IL-10, IL-13 and IFN-γ secretion from stimulated human PBMCs only when complexed with RA. This phenomenon was neither associated with apoptosis of T-cells nor with the activation of Foxp3+ T-cells, but correlated likely with enhanced stability to lysosomal digestion due to a predicted overlap of Cathepsin S cleavage sites with the RA binding site. Taken together, proper loading of Bos d 5 with RA may suppress its immunogenicity and prevent its allergenicity.

## Introduction

IgE-mediated food allergies have an increasing prevalence in industrial countries; up to 10% of the population, both children and adults, are affected by this disease^1^. Important food allergens, including milk, that potentially lead to severe allergic reactions must be labelled on food in the EU. In individuals allergic to food allergens, mast cells in the intestinal mucosa and submucosa release mediators that cause symptoms like enhanced peristalsis, increased fluid secretion from intestinal cells, vomiting and diarrhoea^2^.

Cow’s milk is one of the most important food allergens in children. The onset of symptoms occurs most often during the first year of life when up to 7.5% of infants become affected by milk allergy^3^. In the majority of cases the milk allergy is outgrown by adulthood^4^. However, children who have suffered from milk allergy have a higher risk of developing atopic eczema, egg allergy or allergic asthma^5^, with T-cell driven aspects in the chronic state. Therefore, it is crucial to understand the critical events deciding between tolerance and IgE sensitization, which is predictive for the initiation of clinical disease^6,7^.

Cow’s milk, like human milk, provides nutrition and microelements in addition to important immune defence molecules to the new born. It consists of approximately 35 grams of protein per litre of which 20% is the whey fraction, which mainly consists of proteins with allergenic potency: β-lactoglobulin (Bos d 5), α-lactalbumin (Bos d 4) and serum albumin (Bos d 6). The secretory Bos d 5 represents up to 50% of the whey fractions and 12% of whole cow’s milk protein. In fact, Bos d 5 is (besides Bos d 8, casein) the allergen eliciting most IgE-mediated reactions in allergic individuals^8^. It is a globular protein of 162 amino acid residues with a molecular mass of 18.3 kDa and belongs to the protein family of lipocalins^9^. Lipocalins are functionally diverse and share a low sequence homology, but a highly conserved core structure featuring an antiparallel ß-barrel that defines a calyx^9,10^. The structural similarity amongst lipocalins may cause functional similarity in eliciting – or controlling – allergies, which has to do with their capacity to bind ligands into their calyx^11^. Bos d 5, like other lipocalins, is able to bind small hydrophobic ligands such as fatty acids, vitamin D3 or retinoic acid (RA) suggesting a role as a carrier molecule involved in the innate immune defence, with a potential to influence cellular processes and inhibit bacterial growth by ligand sequestering^12–14^. The binding affinity of the active vitamin A metabolite RA to Bos d 5 ranged from 0.04 to 8.3 µM depending on the analytical techniques and conditions used^15,16^. There is increasing evidence that RA *per se* has an immunomodulatory and anti-inflammatory function^17,18^. Due to its influence on effector Th1/Th2/Th17 cell differentiation and its ability to induce Foxp3 regulatory T cells RA may have an influence on allergic immune responses^19^. Based on these facts we hypothesized that RA, like iron-siderophore complexes in our previous studies^12,20^, could confer immunomodulatory properties to Bos d 5. We therefore investigated whether Bos d 5 could influence immune responses when complexed with the active vitamin A metabolite RA, particularly IgE-binding as well as T-cell responses and the Th1/Th2 dichotomy. If so, the results of this study could have direct implications for industrial production of milk with a lower immunogenic/allergenic potency and affect feeding regimens of dairy milk cows specifically considering vitamin A supplementation^21^.

## Results

### RA binds into Bos d 5 *in silico* and *in vitro*

**Figure 1:**
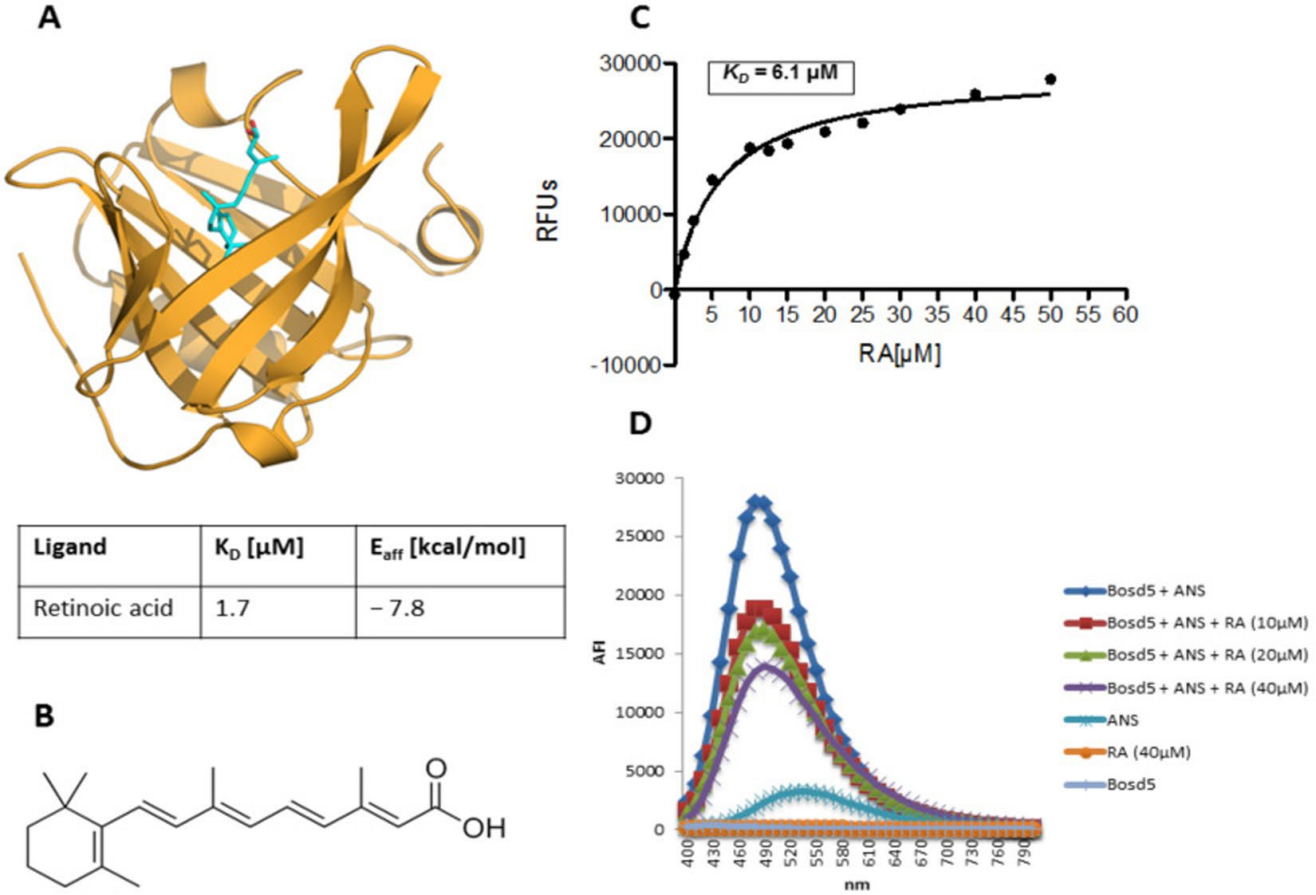
*In silico* and *in vitro* binding of RA to Bos d 5. (**A**) Crystal structure of Bos d 5-RA (turquoise sticks) complex (PDB entry 1GX9); (**B**) structural formula of RA; (**C**) *in vitro* fluorescence spectroscopy of Bos d 5 with increasing concentrations of RA (x-axis in µM); (**D**) *in vitro* ANS competition assay where changes in the fluorescence of ANS signal induced by different molar ratios of Bos d 5 to RA are shown. AFI, average fluorescence intensity.

We performed *in silico* docking analysis using the crystal structure of Bos d 5 (PBD entry 1GX9) and RA (Fig. 1A,B). The best docking solution predicted a complex geometry in complete agreement with the crystal structure (Fig. 1A) and an affinity energy of −7.8 kcal/mol that corresponds to a dissociation constant of 1.7 µM. To confirm the ability of Bos d 5 to bind to RA *in vitro* we used fluorescence spectroscopy (Fig. 1C) and an 1-anilino-8-naphthalene sulfonate (ANS) competition assay (Fig. 1D). In Fig. 1C Bos d 5 was exposed to different concentrations of RA (0 to 50 µM). The complex dissociation constant (*K*_*D*_), resulting from the fluorescence intensity (*F*_0_*/F*_0_ *−* *F)* as a function of the RA concentration, was 6.1 µM, in agreement with Belatik *et al*.^15^.

To affirm the data *in vitro* a ligand competition assay was performed using ANS, an essentially non-fluorescent compound displaying fluorescence only when attached to hydrophobic areas or into a cavity of a protein. Displacement of ANS by ligands such as RA hence results in a decrease of the fluorescent signal. Figure 1D shows that RA dose-dependently (10–40 µM) displaced ANS from Bos d 5, indicating that Bos d 5 is able to bind RA in its hydrophobic calyx.

For both binding assays protein-ligand incubation was done at 4 °C to prevent protein calyx destabilization and degradation, and to promote formation of complexes with the RA ligand, which remain stable even at 37 °C under cell culture conditions^22^. Furthermore, the methods were pivotal to stringently control the ligand loading state when empty Bos d 5 (*apo*-Bos d 5) or Bos d 5 loaded with RA (*holo-*Bos d 5) was used in the experiments below.

### The Bos d 5 ligand RA does not affect its IgE binding capacity

**Figure 2:**
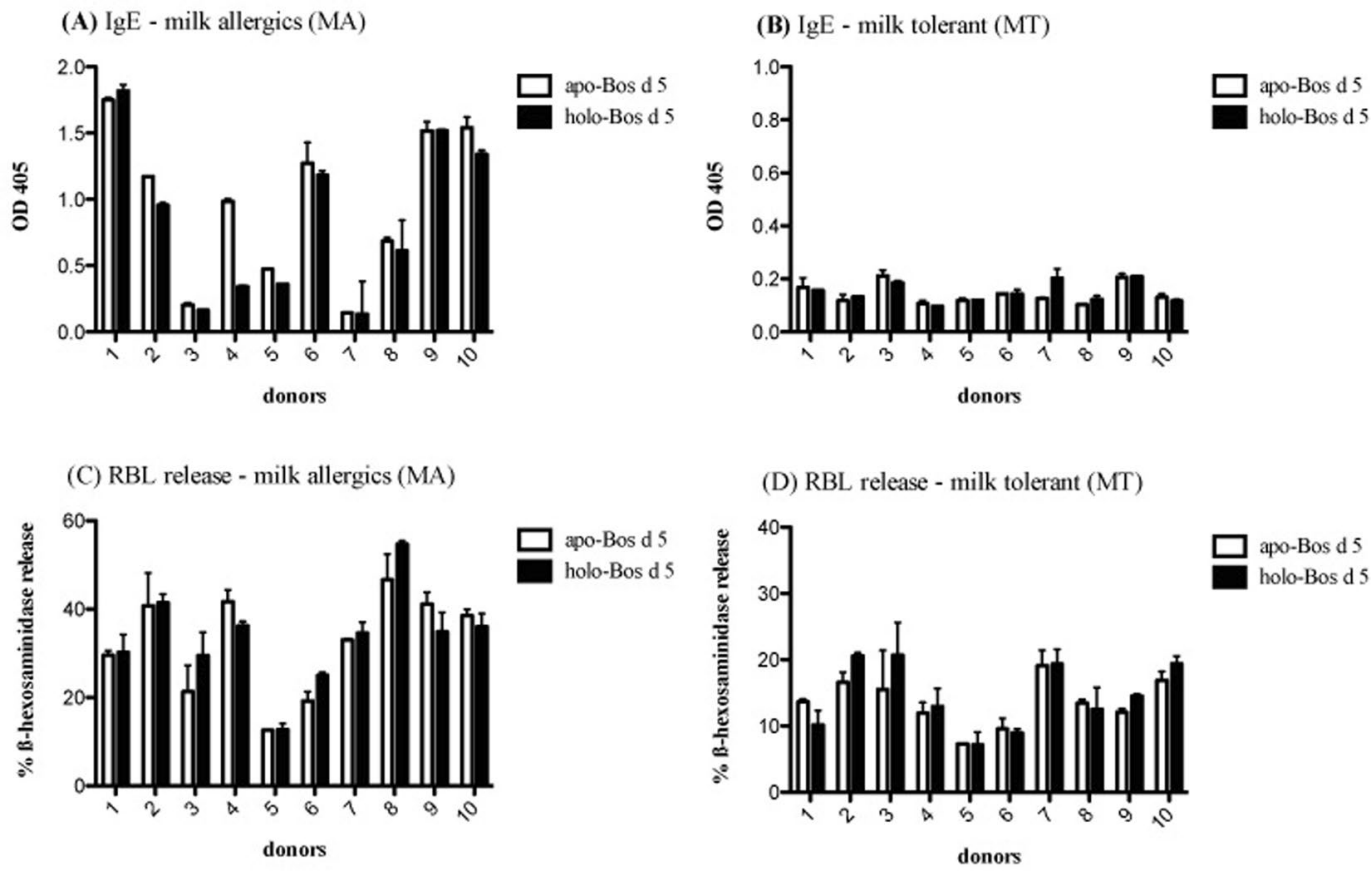
Allergen-specific IgE ELISA (**A**,**B**) and RBL Assay (**C**,**D**). Twenty sera from milk allergic patients were tested for IgE levels (**A**,**B**) and IgE cross-reactivity (**C**,**D**) either with *apo-*Bos d 5 (white bars) or with *holo*-Bos d 5 (black bars). (**A**,**C**) 10 patients with positive reaction to oral cows milk allergen challenge (milk allergic, MA); (**B**,**D**) 10 patients with negative reaction to oral cows milk allergen challenge (milk tolerant, MT). Optical density (OD) was measured at 405 nm.

We determined *apo-* and *holo-*Bos d 5 specific IgE levels in sera from cows milk sensitized children. Ten sera were from allergic patients who reacted positive to oral cows milk allergen challenge (Fig. 2A), termed milk-allergics (MA); the other ten patients did not react clinically to the allergen challenge (Fig. 2B), termed milk tolerant (MT). Eight out of ten MA patients showed similar high specific IgE to *apo- and holo-*Bos d 5, while all MT patients displayed lower specific IgE to *apo*- as well as *holo-*Bos d 5 (Fig. 2). For verification of our ELISA results we performed IgE-mediated mast-cell degranulation *in vitro* using human FcεRI-expressing rat basophil cells after incubation with MA and MT sera. Both *apo*-Bos d 5 as well as *holo-*Bos d 5 did induce similar ß-hexosaminidase release values from RBL cells for milk-allergic and milk-tolerant sera (Fig. 2C,D). The results indicated that complexing of Bos d 5 with RA does not influence the IgE binding capacity of this major cow’s milk allergen.

**Figure 3:**
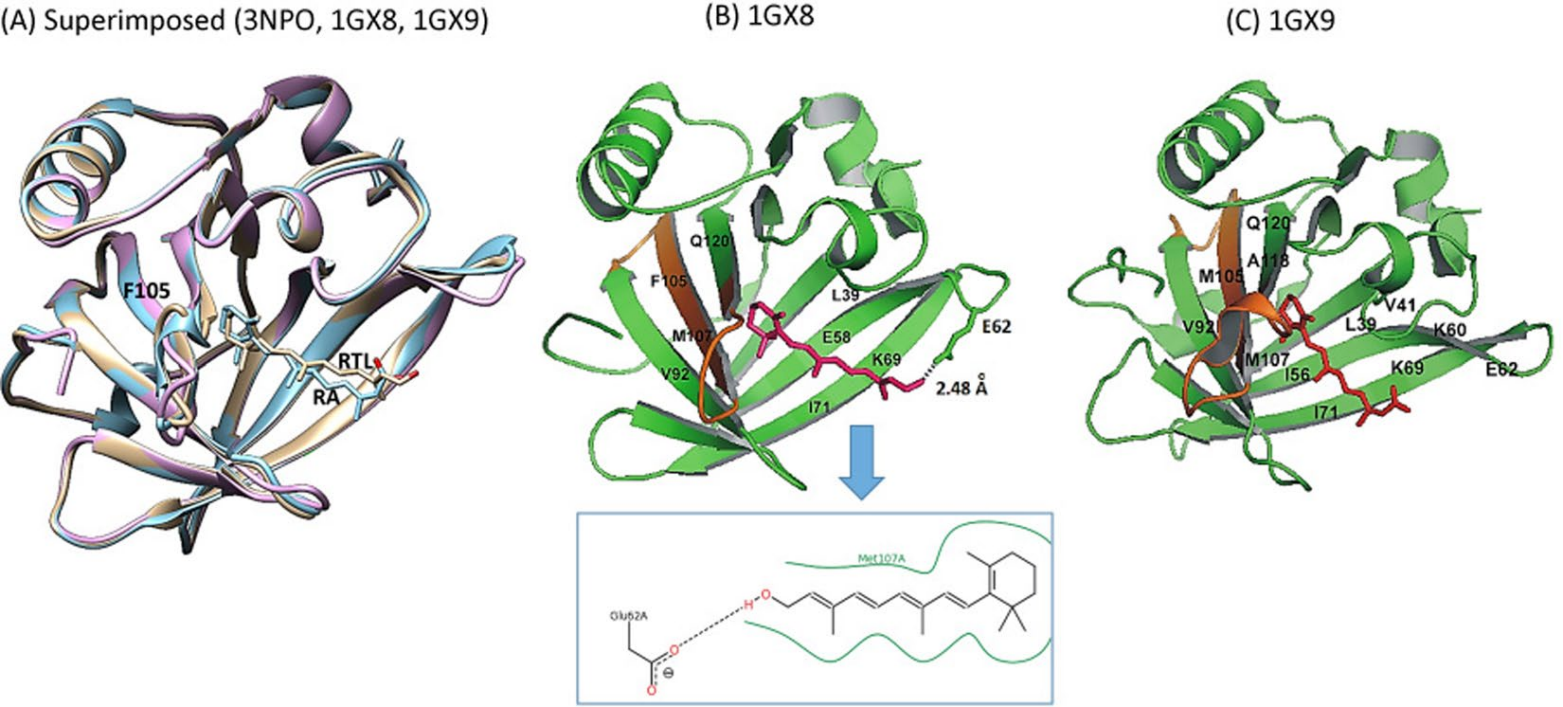
Crystal Structure of free and retinoid-bound Bos d 5 allergen. (**A**) Superimposed *apo-* (3NPO; pink) and *holo-* Bos d 5 structures with retinol (1GX8; copper) and retinoic acid (1GX9; blue) ligands. The two *holo* structures can be superimposed with an over-all main-chain RMSD of 0.39 Å, while the *apo* structure can be superimposed on 1GX8 and 1GX9 with main chain RMSDs of 0.94 Å and 0.98 Å respectively. Positions of retinol (RTL) and retinoic acid (RA) ligands along with the residue F105, which is located in the core region of the T-cell epitope, have been shown. (**B**) and (**C**) Amino acid residues within 4 Å from the ligands retinol (1GX8; 3B) and retinoic acid (1GX9; 3C) in Bos d 5 crystal structures. The ligand retinol is located in close proximity of residue M107 of the T-cell epitope and the side-chain of residue E62 (highlighted in box). E62 is well within distance (2.48 Å) to form a strong hydrogen bond with RTL (1GX8; 3B), whereas it may form a weak hydrogen bond (3.326 Å) with RA (1GX9; 3B). The T-cell epitope region has been shown in orange color in the Bos d 5 structures.

The absence of effect on IgE binding could be explained by addressing the structural basis of the Bos d 5 and RA interactions, and the associated protein-ligand interface. Figure 3A shows the 3-dimensional super-imposed structures of *apo-*Bos d 5 (3NPO in pink), retinol-bound (1GX8 in copper) and RA-bound Bos d 5 (1GX9 in blue), which emphasizes the similarity in the ligand-binding site. Figure 3B,C show interactions in the binding site of the intramolecular pocket between retinol (3B) and RA (3C) and protein residues in the neighbourhood of 4 Å around the ligands. From 12 residues in 3C (L39, V41, I56, K60, E62, K69, I71, V92, F105, M107, A118 and Q120) eight are non-polar and located in the vicinity of the long hydrophobic segment of RA whereas three charged residues are located in the proximity of the carboxylate group of RA, with Glu62 making a weak hydrogen bond with a length of 3.33 Å. Analysis of the retinol-Bos d 5 complex (Fig. 3B) displays the same proportion of non-polar residues as in the RA-Bos d 5 complex, but the number of hydrophobic interactions is lower than those acting on RA. Two charged residues are located in the proximity of the O atom of retinol and E62 shows a hydrogen bond with a length of 2.48 Å, indicating a considerably stronger hydrogen bond interaction than in the case of RA.

Overall, neither RA nor retinol changes the 3-dimensional conformation of Bos d 5. We conclude thus, that the RA loading state of Bos d 5 would have no effect on established immediate type milk allergy in affected patients.

### Retinoic acid binds to the immunodominant T-cell epitope region of Bos d 5

**Figure 4:**
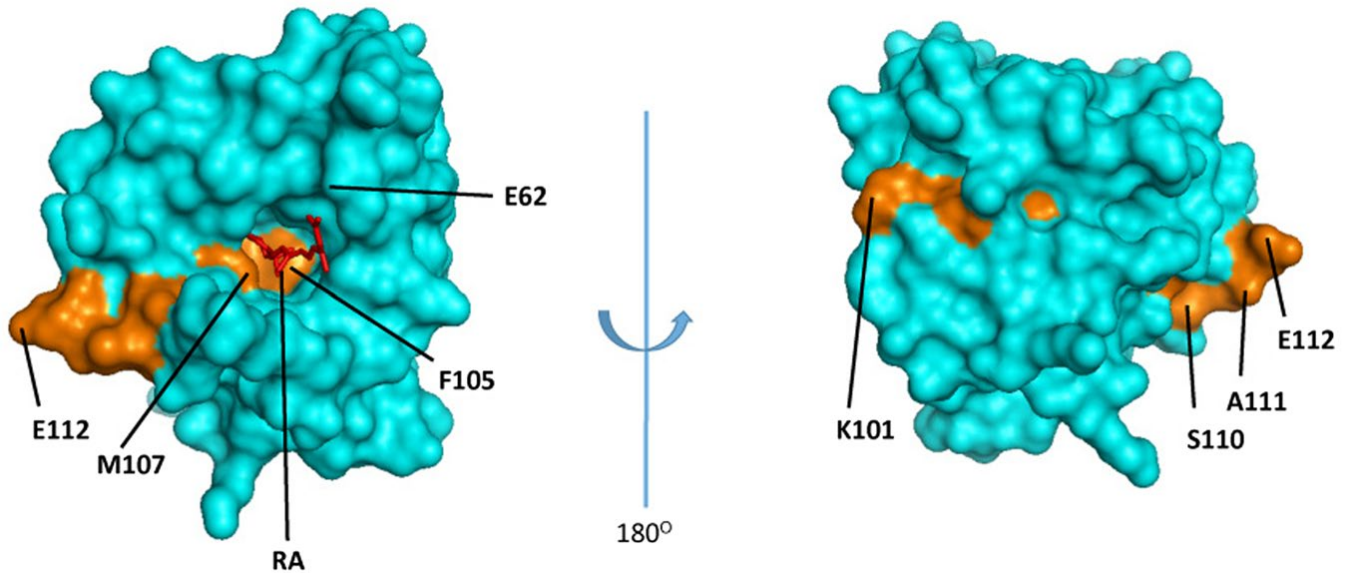
Bos d 5-RA interaction. T-cell epitope residues (K101 to E112) of Bos d 5 are coloured in orange on the Bos d 5 surface, ligand RA is displayed in red. Most residues of the T-cell epitope core region are located in the ligand-binding cavity buried in the structure.

**Figure 5:**
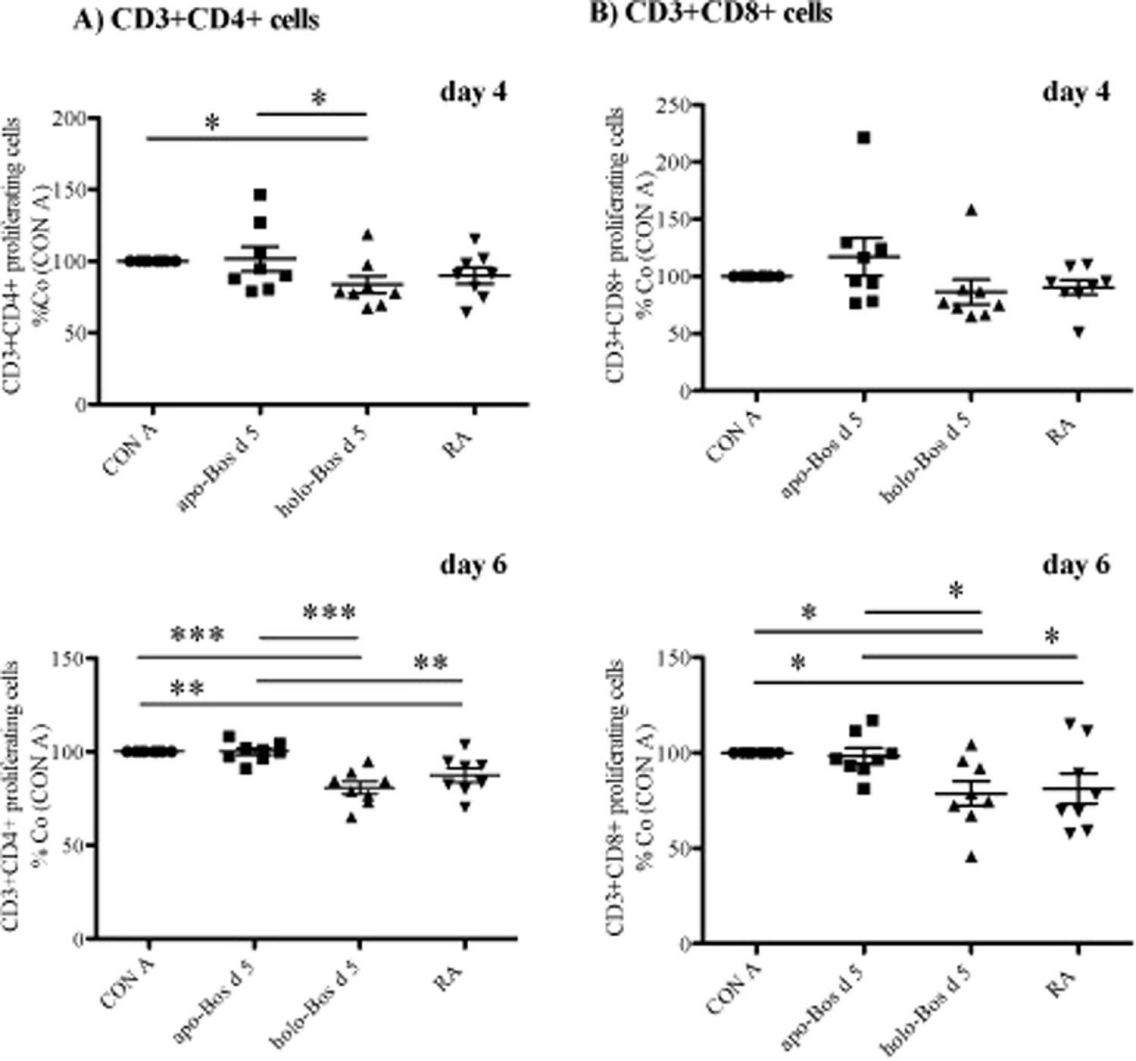
Proliferative response of (**A**) CD3+ CD4+ and (**B**) CD3+ CD8+ positive PBMCs after 4 and 6 days of *in vitro* culture. PBMCs from 8 healthy donors were labeled with CFSE and stimulated with CON A, CON A + *apo-*Bos d 5, CON A + *holo-*Bos d 5 loaded with RA, or with CON A + RA. Proliferative response is shown as percentage of the CON A stimulated control group. X-Axes: substances used for PBMC stimulations; generation of *apo*- and *holo*-Bos d see materials and methods section. Statistical analysis was performed with repeated measures ANOVA following Newman-Keuls Multiple Comparison test. ***p < 0.001, **p < 0.01, *p < 0.05.

Next we explored the potential effect of RA binding in relation to the immunodominant T-cell epitope of Bos d 5 which involves residue numbers 97–117 with the most important core residue K101-E112 (KYLLFCMENSAE)^23,24^. Our calculations predicted this sequence to represent the major portion of Bos d 5 involved in the RA binding (Fig. 3C, Fig. 4). A more recent investigation using single amino acid substitution reconfirmed Y102 to E112 as the minimum essential region on this T-cell epitope region required to elicit T-cell response^25^. Our bioinformatics investigation revealed that protein residues F105 and M107, which are in favourable position to interact with RA (Figs 3C,  4), represent critical T-cell epitope residues of Bos d 5 (Fig. 4). Thus our analysis strongly suggested that RA might modulate the T-cell stimulatory capacity of Bos d 5 via binding to its immunodominant T-cell epitope region. This finding is supported by a significantly reduced proliferative response of human CD3+ CD4+ (p < 0.001) and CD3+ CD8+ (p < 0.05) T-cells when incubated with *holo-*Bos d 5 compared to immune cells stimulated with the empty *apo-*Bos d 5 allergen (Fig. 5, Supplementary Fig. S1).

### Holo-Bos d 5, but not apo-Bos d 5, down-regulates the number of cells expressing CD4+

**Figure 6:**
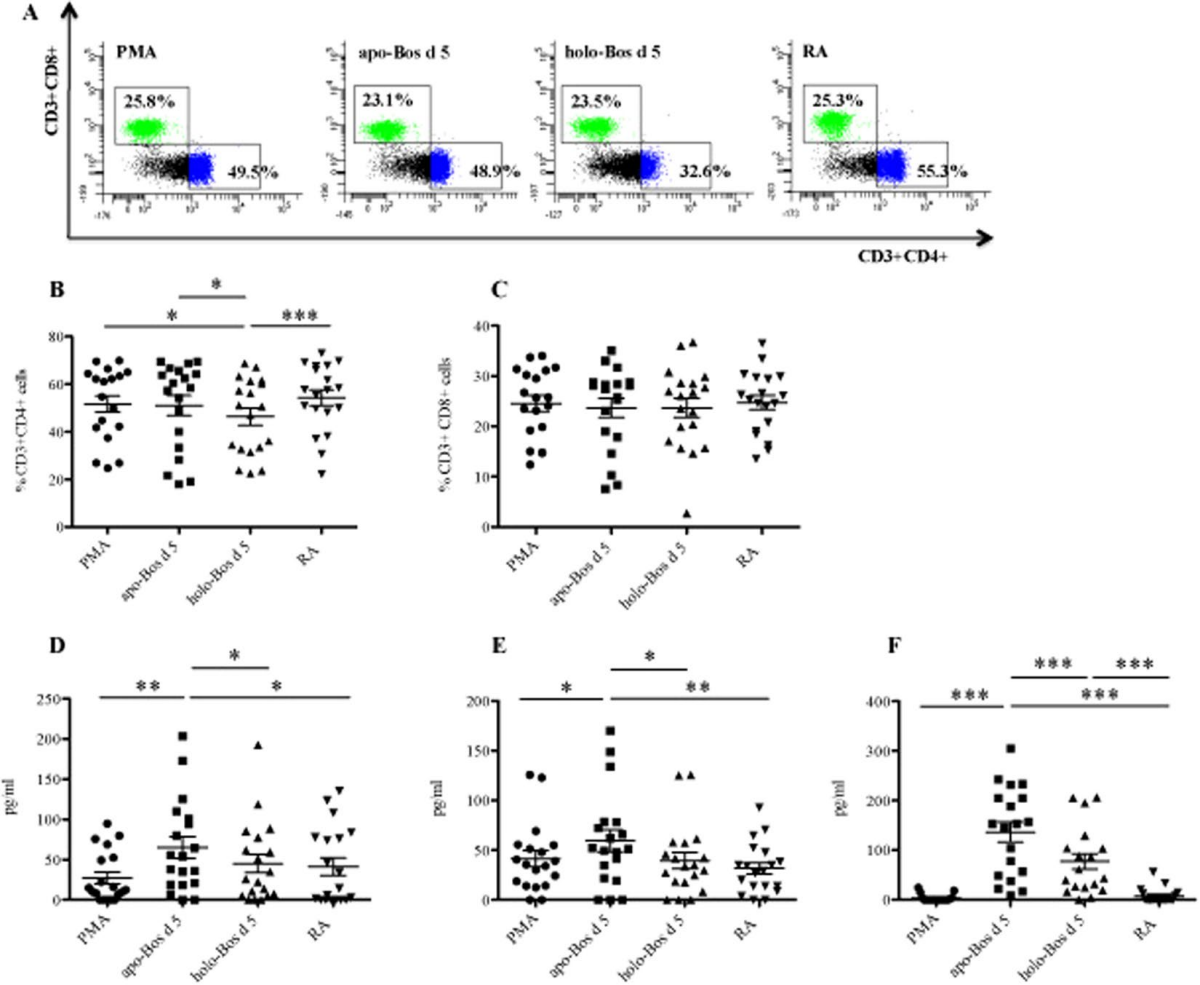
Down-regulation of the number of CD4+ expressing cells and Th1/Th2 cytokines in PBMCs by *holo-*Bos d 5. PBMCs from 19 healthy donors were stimulated with PMA, PMA + *apo-*Bos d 5, PMA + *holo-*Bos d 5, or with PMA + RA. (**A**) Representative pictograms of PBMCs are shown and the percentage of (**B**) CD3+ CD4+ cells and of (**C**) CD3+ CD8+ cells. (**D**) IFN-γ levels (**E**) IL-13 levels and (**F**) IL-10 levels of stimulated PBMCs. X-Axes: substances used for PBMC stimulations; generation of *apo*- and *holo*-Bos d see materials and methods section. Statistical analysis was performed with repeated measures ANOVA following Newman-Keuls Multiple Comparison test. ***p < 0.001, **p < 0.01, *p < 0.05.

Consequently, we concentrated on the T-cell stimulatory capacity of *apo-* versus *holo-*Bos d 5, in analogy to previous studies using siderophore–iron complexes as ligand^12^. We used peripheral mononuclear cells (PBMCs) from healthy individuals and as these cells do not respond to a specific antigen, we used PMA, also known as phorbol 12-myristate 13-acetate, at a low concentration to activate immune cells, and especially T-cells, via protein kinase C^26^. Simultaneously, PBMCs were stimulated for 48 hours with *apo-* or *holo*-Bos d 5, and as a control with RA alone. CD3+ cells were analysed for their CD4+ and CD8+ expression (Fig. 6A). While incubation with the empty *apo*-Bos d 5 did not affect the composition of CD3+ (Supplementary Fig. S2), CD4+ and CD8+ lymphocytes (Fig. 6B,C), we found a significant reduction (p < 0.05) of CD4+ cells after incubation with *holo*-Bos d 5 (Fig. 6B). Notably, *holo*-Bos d 5 had an impact exclusively on the CD4+, but not on the CD3+ (Supplementary Fig. S2) or CD8+ cells (Fig. 6C). This reduction was not due to cell death as neither *apo*-Bos d 5 nor *holo*-Bos d 5 treatments of CD3+ CD4+ lymphocytes resulted in any significantly different expression profile of Annexin V+ cells (Table 1). Treatment with RA alone had no such effect (Fig. 6B,C).

**Table 1:** Percentage of (A) Annexin V positive CD4+ cells and of (B) CD4+ CD25+ Foxp3 positive cells in flow cytometry after stimulations of PBMCs with substances indicated on top.

	PMA	*apo-*Bos d 5	*holo*-Bos d 5	RA
(A) AnnV+	5.95 ± 1.58	5.07 ± 0.84^n.s.^	4.64 ± 1.08^n.s.^	5.92 ± 1.71^n.s.^
(B) Foxp3+	6.60 ± 2.31	6.85 ± 1.90^n.s.^	8.07 ± 2.54^n.s.^	6.44 ± 2.69^n.s.^

PBMCs from 12 healthy donors were treated with PMA, PMA + *apo-*Bos d 5, PMA+ *holo-*Bos d 5, or with PMA + RA. Generation of *apo*- and *holo*-Bos d 5 see materials and methods section. Statistical analyses were done with repeated measures ANOVA following Newman-Keuls Multiple Comparison test. n.s. not significant.

### Apo- but not holo-Bos d 5 differentially induces Th1/Th2 cytokines

We next addressed whether the effects on CD3+ CD4+ T-cells by *holo-* (versus *apo-*) Bos d 5 would have any impact on the Th1/Th2 polarization in terms of cytokine production. Stimulation of PBMCs from 19 healthy donors with empty *apo*-Bos d 5 led to a significant induction of Th1 cytokine IFN-γ (p < 0.01), Th2 cytokine IL-13 (p < 0.05) as well as of the immunoregulatory IL-10 (p < 0.001) (Fig. 6D–F). The treatment with *holo*-Bos d 5 induced a significant reduction of IFN-γ (p < 0.05), IL-13 (p < 0.05) and IL-10 levels (p < 0.001) (Fig. 6D–F). Therefore, *holo-*Bos d 5 suppressed all, Th1, Th2 and immunomodulatory cytokines, while *apo-*Bos d 5 induced them and thus was more immunogenic. Treatment with RA alone had no such effect, underlining the importance of complex formation between Bos d 5 and RA (Fig. 6D–F).

As RA, together with the regulatory cytokine TGF-ß1, is one of the critical factors that provides signals for differentiation of Foxp3+ T regulatory cells^19^, we aimed to determine any capacity of *holo*-Bos d 5 or *apo*-Bos d 5 to induce regulatory T-cells. PMA-activated PBMCs comprised 6.6% CD4+ CD25+ Foxp3+ cells and stimulations with neither *holo*- nor *apo-*Bos d 5 revealed any statistically significant difference (Table 1). In addition we analysed the endogenous TGF-ß1 expression in cell culture supernatants. Most donors displayed TGF-ß1 levels close to the detection limit of the assay and we found no statistically different TGF-ß1 expression levels in *apo-*Bos d 5 and in *holo-*Bos d 5 stimulated cells. Only treatment with RA alone significantly increased TGF-ß1 levels (Supplementary Fig. S3).

### Influence of RA binding on fold conformation and stability of Bos d 5

**Figure 7:**
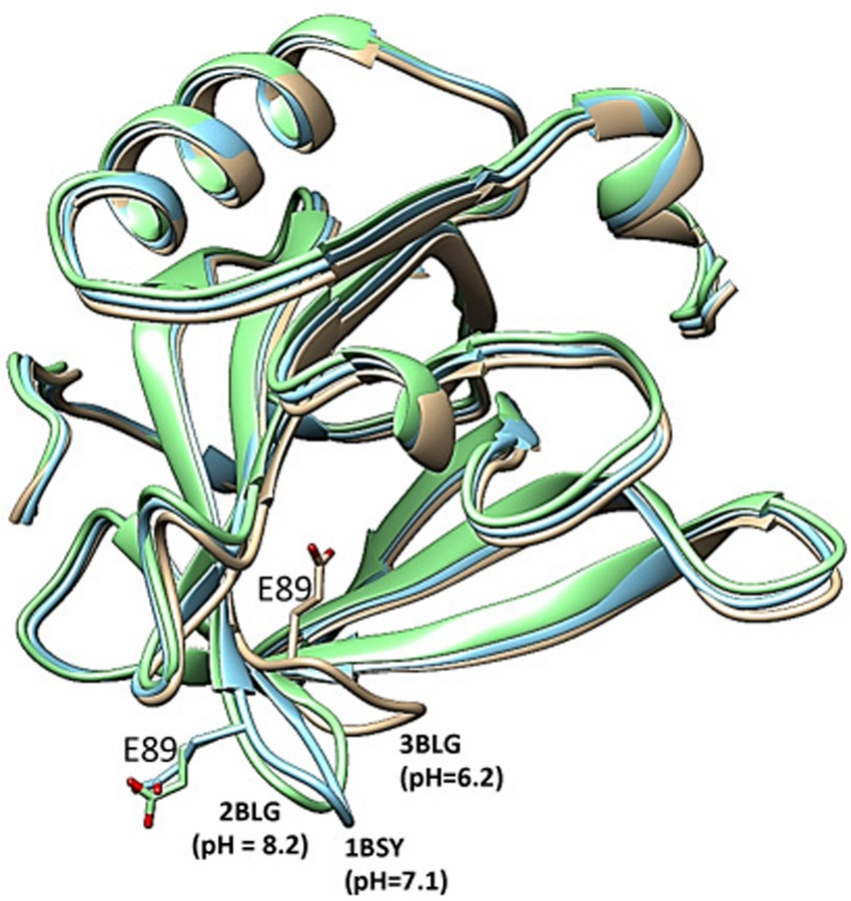
Effect of Tanford transition. Superimposition of Bos d 5 crystal structures at pH 6.2 (3BLG; copper), pH 7.1 (1BSY; blue) and pH 8.2 (2BLG; green) shows the pH-dependent conformational change of the ‘EF-loop’ region (D85 to N90). The flipping of E89 residue at pH 6.2 restricts the entry and loading of ligands, thereby modulating the allergenic properties of Bos d 5.

Previous *in vitro* studies have shown that Bos d 5 binds to retinoids in a pH range of 6.5–8.5^27^; therefore pH could modulate its immunogenicity. Studies on pH-induced conformational changes of Bos d 5 revealed that a conformational change (known as Tanford transition) occurs between pH 6.3 to 8.2, which predominantly involves its ‘EF-loop’ (residues 85–90) region^28,29^. Crystallographically solved structure of this protein at pH 6.2, 7.1 and 8.2 showed that its hydrophobic cavity remains open at pH 8.2 and 7.1, but is closed by the EF loop at pH 6.2 or lower, which makes the hydrophobic core inaccessible to ligands^30^. The ribbon diagram is shown in a triple optimized superimposition (1BSY in blue, 2BLG in green, 3BLG in copper) with the pH-dependent change in key residue E89 important for its immunologic properties (Fig. 7). The functional/physiological implications of this pH-controlled access to the calyx are a protection of stably bound ligands inside the calyx in an acidic environment (e.g. acidic stomach). Thus, environmental pH can control retinoid loading through Tanford transition, thereby influencing the immunogenic and allergenic properties of Bos d 5.

Endolysosomal enzymes play a key role in antigen processing and presentation by generating antigenic peptides^31^. While a high concentration of MHC-II –bound peptide on the cell surface favours Th1-like responses, a lower density promotes Th2 responses^32^. Among endolysosomal proteases, cathepsin S is predominantly expressed in antigen presenting cells and displays a pH optimum of 6.0 to 7.5^33^. It plays an important role in early antigen processing with higher activity in the endosome, compared to the lysosomal compartments^34^. A recent study demonstrated that most of the peptides derived from major birch pollen allergen Bet v 1, could be generated by the endolysosomal protease cathepsin S alone, and had effects on antigen presentation^35^.

In a preliminary analysis the online probability score-based protease cleavage site prediction tool (http://lightning.med.monash.edu/prosperous/;accessed December 15, 2016) indicated a possible overlap of Cathepsin S cleavage sites with the RA binding site in Bos d 5 (Supplementary Fig. S4 A,B). Thus, the accessibility to the protease cleavage site could be influenced by RA binding and already in the lysosomes during antigen processing. Consequently, this would lead to an antigenic masking of the immunodominant T-cell epitope (Fig. 4, Supplementary Fig. S4) and prevent the immunogenicity of the milk allergen Bos d 5.

## Discussion

Bos d 5, one of the major allergens in cow’s milk, belongs to the lipocalin protein family. Due to structural similarity, Bos d 5 has also been classified as a member of the retinol binding protein-like class^14^. Retinol and its metabolites such as RA are present in bovine milk and can be transported via Bos d 5^36,37^. In accordance with previous reports^38^ our *in silico* docking analysis predicted a high binding affinity of the milk allergen to RA, as suggested by an estimated K_D_ of 1.7 µM. This prompted us to further address the binding affinity by two different *in vitro* approaches. First we used the principle of quenching the intrinsic fluorescence of Bos d 5 as an indicator for ligand binding, resulting in a K_D_ of 6.1 µM, in agreement with the order of magnitude predicted by docking calculations although not fully achieving the binding estimated *in silico*. Accordingly, other groups have reported similar results using fluorescence quenching^15^. For a final demonstration of the high binding affinity of Bos d 5 to RA, we adapted an ANS competition assay showing a dose-dependent decrease of fluorescence signal with increasing ligand-protein ratio^39^. Our data clearly confirm the ability of Bos d 5 to bind RA. More importantly, these techniques were crucial to precisely document the ligand loading state of Bos d 5 in all subsequent experiments.

Clinical and immunological relevance of ligand binding to allergenic proteins has recently been emphasized^12,20,40^, but mechanisms by which ligands modulate immune-reactivity are not fully understood. In fact, binding of RA had no influence on the IgE binding and cross-linking capacity in sera of children sensitized to milk allergen, being tolerant (MT) or reacting positive to oral cows milk allergen challenge (MA). The human IgE-Bos d 5 interaction has been crystallographically elucidated recently^41^. Considering human IgE epitope information on Bos d 5, two experimentally deduced regions (K75-D85 in the loop and E127-P144 in the α-helix region), with three associated ‘weaker’ stretches (L31-P48, K47-K60, and L57-I78) were described to bind patient IgE^42^, but none of these residues seems to overlap with the RA binding site (Fig. 4). In addition, analysis of the epitope-paratope interface in the Bos d 5–human IgE co-crystal structure (2R56) showed that this conformational IgE epitope involves six different short stretches of residues covering a flat area on the Bos d 5 surface^41^, also not involved in RA binding.

Our analysis confirms that RA binding to Bos d 5 has no significant impact on its 3D-conformation that can alter its antigenic surface and thereby disrupt IgE binding. We therefore concluded that RA-loading of the milk allergen would have no impact on already established milk allergy, but might rather have immunologic impact, for instance on its T-cell stimulatory capacity and cytokine production.

Our subsequent bioinformatics analysis revealed that the main part of the Bos d 5-RA interface involved in RA binding, is critically overlapping with the major T-cell epitope of Bos d 5^23,25^. Studies in healthy and allergic Asian cohorts suggested a high affinity of this Bos d 5 epitope to the HLA-DRB1*0405 allele^24^. A preliminary analysis of a small sample of our study population, all Caucasians, did not reveal this specific allele (see Supplementary Table S1, supplementary methods). It has been reported that HLA types are distinct in different ethnic groups^23^ and a recent publication describes that also other alleles can have a high affinity to milk allergens and therefore be susceptible to cows milk allergy^43^.

We then compared the stimulatory capacity of *apo*- versus *holo*- Bos d 5 on human primary immune cells *in vitro*; unexpectedly, stimulation with *holo*-Bos d 5 led to a significant decrease in the CD3+ CD4+ cell subset, compared to cells exposed to *apo*-Bos d 5, indicating an immunosuppressive effect on this population which is pivotal in allergy induction^44^. Additionally, CD3+ CD4+ T-cells showed a time-dependent significant reduction of proliferative response after stimulation with *holo*-Bos d 5 when compared to the treatment with the empty *apo*-protein. On the cytokine level *apo*-Bos d 5 induced a mixed Th1/Th2 profile with high IFN-y, IL-13 and IL-10 similar to results from a previous study of our group^12^. In contrast, stimulation with *holo-*Bos d 5, but not with RA alone, resulted in a significant decrease of all three cytokines. In a mixed PBMC population, as utilized in our study, cytokines could also be derived from other immune cells besides Th2 lymphocytes, such as Th1 (IFN-γ) or B regulatory cells (IL-10)^45^. In fact, paralleling Th1 and Th2 responses have been described before^46^. The most significant finding, however, was that *holo*-Bos d 5 impeded any of the investigated immune cell activation. Interestingly, other lipocalin proteins in connection with small hydrophobic ligands have been described to act in a similar manner^40,47^. The *holo*-Bos d 5 mediated effects were thus comparable to the events occurring in the development of natural tolerance to milk and other food allergens^7^.

We suspected apoptosis as a relevant underlying mechanism for the loss of the CD3+ CD4+ cell+ population but the expression profile of AnnexinV+ CD4+ cells revealed no significant differences after treatment with *apo*- or *holo*-Bos d 5. These data implied that the decrease of CD3+ CD4+ cells indeed derived from the immunosuppressive function of *holo*-Bos d 5 by down-regulating CD4+ expression, possibly before signs of apoptosis could be detected^12^. RA *per se* can influence cell viability but the concentration used in this experiment was within a physiological range with concentrations between 1 and 1000 nM causing no damage to human T cells^48^. As we worked with PBMCs, already at this time point, we could not exclude a differential antigen processing of Bos d 5 depending on the ligand load.

Since clinical studies pointed out that resolution of milk allergy was associated with a higher frequency of regulatory T cells in PBMCs of milk tolerant subjects^49,50^, we investigated the effects of *apo-* or *holo-*Bos d 5 stimulations on the compartment of Foxp3 positive cells in the CD4+ CD25+ T cell population, but found no significant changes. Also RA alone showed no effect, as it usually only in synergy with TGF-ß or rapamycin is able to enhance Foxp3 expression in human T cells^51,52^.

So far, our data revealed that RA binding into Bos d 5 had no impact on IgE binding, but profound inhibitory effects on its immunogenicity, likely by binding and masking the major T-cell epitope of this milk allergen. We asked whether RA binding could potentially have an effect on the fold stability of Bos d 5, and on the fate of the T-cell epitope during the antigen processing. Recent studies documented an association between resistance to proteolytic digestion and allergenicity of ingested proteins^53,54^, and it was shown that binding of retinoids and other vitamin ligands could increase the stability and protease resistance of Bos d 5^55,56^. Fold stability can determine the sensitizing potential of allergens by altering epitope specific T-cell activation which was demonstrated for the major birch pollen allergen Bet v 1 in two recent studies^57,58^. We speculated that RA binding could also stabilize the Bos d 5 fold structure and reduce protease accessibility. Further, our preliminary *in silico* analysis predicted RA to attach to a major cleavage site of the endolysosomal enzyme cathepsin S. This, together with the masking of the major T-cell epitope by RA, could substantially minimize its immunogenicity and thereby prevent allergies.

In conclusion, our study revealed that Bos d 5 is able to bind RA in its calyx with high affinity and that there are distinct immunomodulatory effects of the empty (*apo*-) and loaded (*holo*-) form of Bos d 5 on human primary immune cells. These findings contribute to the understanding of the mechanism of the Th2 bias in allergic sensitisation and utilize knowhow from our previous studies on other lipocalin allergens. Our results suggest that Bos d 5, when loaded with RA *(holo-)* is able to prevent an immune response, leaving the CD3+ CD4+ cell population and Th2 related cytokines unresponsive. Due to the fact that RA alone did not show this effect on T cell subsets and cytokine expression, we propose that the immunosuppressive phenomenon truly derives from the Bos d 5/RA complex. In contrast, *apo-*Bos d 5 stimulates this population and has a clear immunogenic effect. Therefore, proper loading of this major cow’s milk allergen may prevent subsequent allergic immune responses to it.

Milk is pivotal in human nutrition but may, due to loss of lipophilic ligands such as RA, gain immunogenicity and allergenicity during large-scale production. To prevent this we propose the following: i) The feeding of cows with organic food containing RA is substantial for Bos d 5 loading to keep milk tolerogenic; ii) In dairy industry, during defatting processes natural hydrophobic compounds including RA are lost and have later to be supplemented, but precise loading of RA into the Bos d 5 pocket seems to be critical; iii) high performance milk production by “turbo cows” may result in insufficient loading of Bos d 5^59^, turning a harmless nutritional protein into a milk allergen.

## Material and Methods

### Materials

Bovine beta lactoglobulin (Bos d 5), all *trans*-retinoic acid (RA), deferoxamine mesylate, phorbol 12-myrisate 13-acetate and Concanavalin A (CON A) were purchased from Sigma (Sigma Aldrich, Steinheim, Germany). Ficoll-Paque PLUS was from GE Healthcare (Uppsala, Sweden). 5(6)-Carboxyfluorescein diacetate N-succinimidyl ester (CFSE) and ELISA kits for IL10, IL13, IFNy and TGF-ß1 were obtained from eBioscience (Santa Clara, CA, USA). FACS antibody CD3-APC clone SK7 and 7AAD were purchased from eBioscience, antibodies CD4-PE-Cy7 clone SK3, α8-PE clone SK1, and Annexin V FITC were from BD Biosciences (San Jose, CA, USA). Human regulatory T cell staining kit was obtained from eBioscience.

### *In silico* docking analysis

The geometry of Bos d 5 was taken from the crystal structure of its complex with RA (protein data bank (PDB) entry 1GX9)^12^. AutoDockTools was used to prepare input structure files for protein and ligand. Docking calculations were performed with AutoDock Vina^60^. The lowest affinity energy (E_aff_) (best docking solution) was used to estimate the dissociation constant K_D_ for the protein-ligand complexes under the assumption E_aff_ ~ΔG with K_D_ = exp(−ΔG/RT) at *T* = 298.15 K^12^.

### Structural bioinformatics analysis

Protein structural diagrams were prepared by UCSF Chimera and Pymol (The PyMOL Molecular Graphics System, Version 1.8 Schrödinger, LLC, 2015^61,62^). Surface area, solvation energy and solvation effect calculations were performed using PISA module of CCP4 package^63^. Ligand Explorer was used to analyse the ligand-protein interaction and to measure distances, which was further validated by NCONT module of CCP4^64^. For protease cleavage site prediction PROSPERous server was used under http://lightning.med.monash.edu/prosperous/ (“PROSPERous: a comprehensive server for predicting protease-specific substrates and cleavage sites using a combination of multiple scoring functions. Submitted for publication”)^65^.

### Generation of apo- and holo-Bos d 5

Commercially available bovine beta-lactoglobulin (L0130, Sigma) was dissolved in deionised water (20 mg/m) and dialyzed three-times against 10 µM deferoxamine mesylate salt. Further dialyzation against deionized water was performed (*apo-*Bos d 5). *Holo*-Bos d 5 for *in vitro* stimulations was generated by pre-incubation of 2.5 µM *apo-*Bos d 5 as described above with 1 µM RA dissolved in dimethyl sulfoxide (DMSO).

Analysis of Bos d 5 preparations by SDS-PAGE-gel revealed a predominant band at 18 kD without evidence for the presence of substantial amounts of other milk proteins (see Supplementary Figure 5, lane 3). Lipopolysaccharide (LPS) concentration of Bos d 5 was 1,996 EU/ml (concentration 1 mg/ml)^12,49^. RA, PMA and CON A did not contain substantial amounts of LPS (<0.122 EU/ml).

### Bos d 5 - RA binding Studies

#### Fluorescence quenching assay

Stock solutions of Bos d 5 (1 mM) and RA (100 µM) were prepared in Tris-buffer at pH 7.4. Serial dilutions of RA (0–50 µM) were incubated with 7.5 µM Bos d 5 for 30 minutes at 4 °C. DMSO, RA and buffer served as controls. The fluorescence spectra were recorded at λ_exc_ = 280 nm and λ_emi_ = 310 to 500 nm. The intensity at 340 nm (tryptophan) was used to calculate the binding constant (K_D_) of the fluorescence intensity (F_0_/F_0_ − F) as a function of RA concentrations.

#### ANS competition assay

Bos d 5 (100 µM final concentration) was incubated with RA (10, 20, 40 µM) in 96-well UV-Star plates (Thermo Fisher Scientific, Waltham, MA, USA) in different ligand-to-protein molar ratios (1:2.5–1:10) overnight at 4 °C. Prior to measurement 5 µL ANS (50 µM final concentration) was added and the mixtures were incubated for another hour at room temperature. Controls consisted of RA, Bos d 5, ANS and DMSO alone as well as Bos d 5 with ANS. Absorbance spectra were recorded at λ_exc_ = 350 nm and λ_emi_ = 300 to 800 nm in 10 nm steps. Read-outs for both assays were performed on a TECAN Plate Reader Infinite M200 PRO.

### Allergen-specific IgE ELISA

For detection of Bos d 5-specific IgE in sera of milk allergic patients (10 patients positive and 10 patients negative to oral cows milk allergen challenge; sera were retrospectively collected in accordance with the Helsinki Declaration of 1975 and under approval of the ethical committee of the Bambino Gesù Pediatric Hospital, Rome; individual informed consent from all donors was collected by Dr. Alessandro Fiocchi, Children’s hospital Bambino Gesú, Rome, Italy), duplicate wells of MaxiSorp 96 well flat-bottom plates (Nunc, Rochester, NY, USA) were coated with 100 µl/well of *apo-*Bos d 5 or *holo-*Bos d 5 diluted at 5 µg/ml in coating buffer and incubated overnight at 4 °C. After 2 h blocking at room temperature with 200 µl Tris buffered saline (TBS)+ 0.05% Tween 20 (TBST), wells were incubated with 100 µl of human serum diluted 1:5 in TBST overnight at 4 °C. Alkaline-phosphatase (AP) conjugated mouse anti-human IgE antibody (BD Pharmingen) diluted at 1:1000 in TBST was used as secondary antibody. AP substrate (100 µl/well; SIGMAFAST p-Nitrophenyl phosphate Tablets, Sigma) was added to detect bound AP conjugated antibodies. The optical density was measured at 405 nm using an Infinite M200Pro microplate reader (Tecan, Austria).

### RBL degranulation assay

Human FcεRI-expressing rat basophil cells (RBL-SX38) (2 × 10^4^/well in 96 well plates) were sensitized overnight with human serum of milk allergic patients (see ELISA), 1:10 diluted in Tyrode’s buffer. Sensitized cells were stimulated with 1 µg/ml *apo*-Bos d 5 or *holo*-Bos d 5 in Tyrode’s buffer for 1 h at 37 °C (protein concentration inducing highest degranulation determined by dose–response experiments). Degranulation was assessed by measurement of released ß-hexosaminidase in the supernatant and of unreleased enzyme in the respective cell lysate. The presented results were calculated as percentage release of total ß-hexosaminidase content.

### Isolation of PBMCs

The study was approved by the institutional ethics committee of the Medical University of Vienna and conducted in accordance with the Helsinki Declaration of 1975. Nineteen healthy volunteers donated 15 mL blood. All subjects gave their fully written informed consent (EK Number 1146/2013, Medical University of Vienna).

Heparin-treated blood was mixed with equal volumes of PBS containing 2% fetal calf serum (FCS) before applying 10 mL Ficoll-Paque PLUS and centrifuged at 400 g for 30 minutes without brake. After density gradient separation the lymphocyte fraction was isolated and transferred to a fresh tube. Cells were washed twice with 0.9% sodium chloride solution. Subsequently, cells were diluted to a concentration of 1 × 10^6^ cells/mL in RPMI medium containing 10% FCS, 2 mM L-glutamine and 1% penicillin/streptomycin.

### Stimulation of PBMCs for CD marker expression and cytokine analysis

Based on previous titration experiments^12^, we used 2.5 µM (50 µg/ml) of *apo*-Bos d 5 for *in vitro* stimulation. *Holo*-Bos d 5 (see above) and RA concentration resulted from preliminary titration experiments that were essential to determine RA dosage especially for *in vitro* control groups. For RA control groups 1 µM was used.

Isolated PBMCs (0.5 × 10^6^/well) were incubated with a final concentration of 0.25 ng/mL PMA, PMA + *apo*-Bos d 5, PMA+ *holo-*Bos d 5 or PMA+ RA. The cells were incubated for 48 hours at 37 °C. After 48 hours supernatants were collected and stored at −80 °C until further analysis. Cells were stained for 30 minutes at 4 °C with CD3-APC, CD4-PE-Cy7 and CD8-PE in PBS containing 2% FCS followed by 10 minutes incubation with AnnexinV FITC in binding buffer (10 mM HEPES, 140 mM NaCl, 2.5 mM CaCl_2_) at room temperature. We gated on living cells by forward and sideward scatter. Further gating was done on CD3 cells, before plotting towards CD4 and CD8 positive cells. Due to the down-regulation of the surface CD4 expression on CD4+ cells upon PMA stimulation^26^ we used control cells that were cultured under the same conditions but without treatment of PMA and the CD4+ positive gate was set according to these controls. CD4+ CD25+ Foxp3+ T regulatory cells were stained according to the manufacturer’s protocol with CD4-FITC, CD25-APC and Foxp3-PE or Foxp3-PE isotype control. Acquisition and analysis was performed on a FACS Canto II machine (BD Bioscience, San Jose, CA, USA) using the FACSDiva Software 6.0.

### CFSE cell proliferation assay

PBMC proliferation was determined using CFSE according to previously published protocols^66^. Briefly, CFSE at a final concentration of 4 µM (1 mM stock solution) was added to 1 × 10^7^ PBMCs for 10 minutes at room temperature. Subsequently cells were washed twice with RPMI containing 10% FCS. CFSE stained cells (1 × 10^5^/well) were incubated in 96-well plates with CON A (2 µg/ml), CON A + *apo-*Bos d 5, CON A+ *holo-*Bos d 5 and CON A+ RA for 4 or 6 days (concentrations of *apo*-, *holo*-Bos d 5 and RA as described above); CFSE dilution and T-cell population was determined by flow cytometry using CD3-APC and CD4-PE-Cy7 antibodies. For gating strategy and representative histograms see supplementary Figure S1. Acquisition and analysis was performed on a FACS Canto II machine (BD Bioscience, San Jose, CA, USA) using the FACSDiva Software 6.0.

### Determination of cytokines

IFNy, IL-10, IL-13 and TGF-ß1 were detected with commercially available kits according to the manufacturer’s protocol. IFNy, IL-10 and IL-13 have a reported sensitivity of 4 pg/mL, TGF-ß1 of 8 pg/mL.

### Statistical analysis

Statistical analyses was performed with repeated measures ANOVA following Newman-Keuls Multiple Comparison test using GraphPad Prism 6 software (GraphPad, San Diego, CA, USA). p < 0.05 was considered statistically significant.

## Electronic supplementary material

Supplementary Information
