# Supplementary material for: Retinoic acid prevents immunogenicity of milk lipocalin Bos d 5 through binding to its immunodominant T-cell epitope
Source: Sci Rep. 2018 Jan 25;8:1598. doi: 10.1038/s41598-018-19883-0 (PMC5785490; doi:10.1038/s41598-018-19883-0)
Supplement: Supplementary file 1 — Supplementary Information [file 41598_2018_19883_MOESM1_ESM.pdf]

Supplementary Information

Retinoic acid prevents immunogenicity of milk lipocalin Bos d 5 through binding to its immunodominant T-cell epitope

Karin Hufnagl, PhD[a], Debajyoti Ghosh, PhD[b] Stefanie Wagner, M.Sc.[a], Alessandro Fiocchi, MD[c], Lamia Dahdah, MD[c], Rodolfo Bianchini, PhD[a], Nina Braun, B. Sc.[a], Ralf Steinborn, PhD[d], Martin Hofer, B.Sc.[d], Marion Blaschitz, PhD[e], Georg A. Roth, MD[f], Gerlinde Hofstetter, M.Sc[a], Franziska Roth-Walter, PhD[a], Luis F. Pacios, PhD[g], Erika Jensen-Jarolim, MD[a,h]

Supplementary materials and methods

HLA-DRB1 loci sequencing

Five representative donors from our study cohort donated epithelial cells from the inside of the cheek taken by a buccal brush (Celetta brush; Frisetta Kunststoff GmbH, Schönau im Schwarzwald, Germany). DNA was isolated with a QIAamp DNA Mini Kit (QIAGEN) according to the manufacturers instructions. DNA amplification, purification and Sanger sequencing of HLA alleles from DNA were done using a SeCore® DRB1 Locus Exon 2 & 3 sequencing kit (Thermo Fisher Scientific) according to the manufacturers instructions[1]. Capillary electrophoresis of PCR products was conducted on a genetic analyzer 3500 DX (Applied Biosystems) and uType 7.0 sequencing software (OneLambda) was used for analysis.

1. Liu C, Yang X, Duffy B, Mohanakumar T, Mitra R et al. ATHLATES: accurate typing of human leukocyte antigen through exome sequencing. *Nucleic Acids Research* 2013, 41 (14):e142. doi: 10.1093/nar/gkt481. Epub 2013 Jun 8.

Supplementary Figure S1: Proliferative response - histograms of CFSE labelled (A) total PBMCs, (B) CD3+ T-cells, (C) CD3+CD4+ T-cells and (D) CD3+CD8+ T-cells from one representative donor after 4 days in culture. CFSE stained cells (1×10^5/well) were incubated in 96-well plates with CON A (termed Medium), CON A + RA (termed RA), CON A + *apo*-Bos d 5 (termed Bos d 5) and CON A + *holo*-Bos d 5 (termed Bos d 5 + RA) for 4 days. Percentage of the proliferative cells is indicated in each histogram.

Gating strategy: gating was done on living lymphocytes based on the forward and side scatter, excluding dead cells and monocytes. Cell duplicates were removed by comparing the height and area of the forward scatter signal. Lymphocyte singlets were gated on CD3+ and divided into CD3+CD4+ and CD3+CD4- (corresponding to CD3+CD8+).

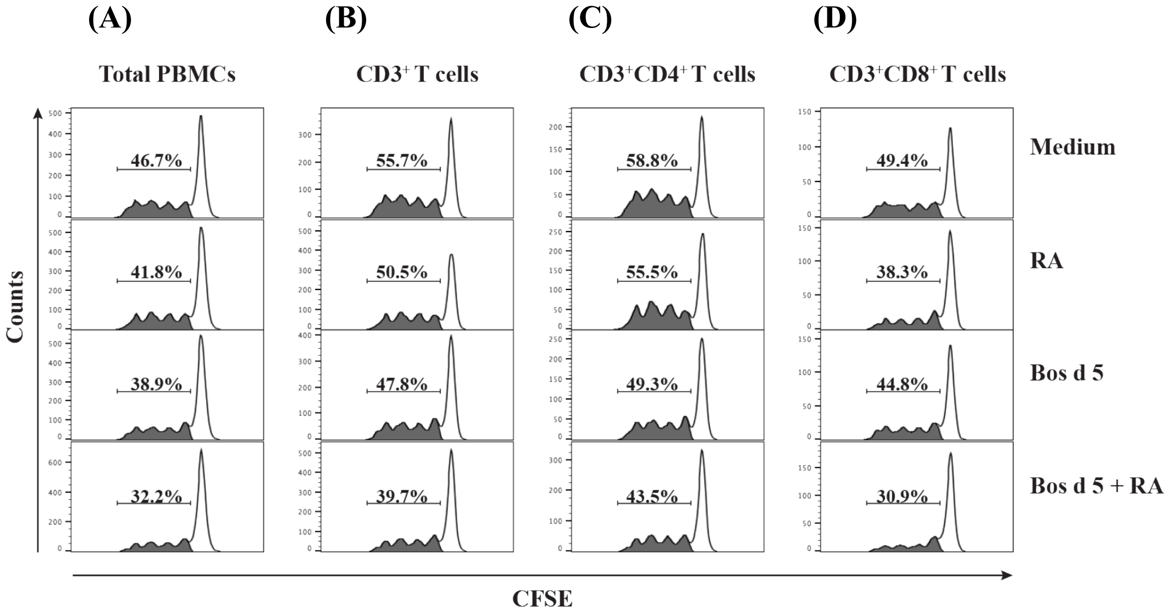

Supplementary Figure S2: Percentage of CD3+ cells. PBMCs of 19 healthy donors were treated with PMA alone, PMA + *apo*-Bos d 5, PMA + *holo*-Bos d 5 loaded with RA, or with PMA + RA for 48 hours. Statistical analysis was performed with repeated measures ANOVA following Newman-Keuls Multiple Comparison test. Statistical analysis did not reveal any significance between groups.

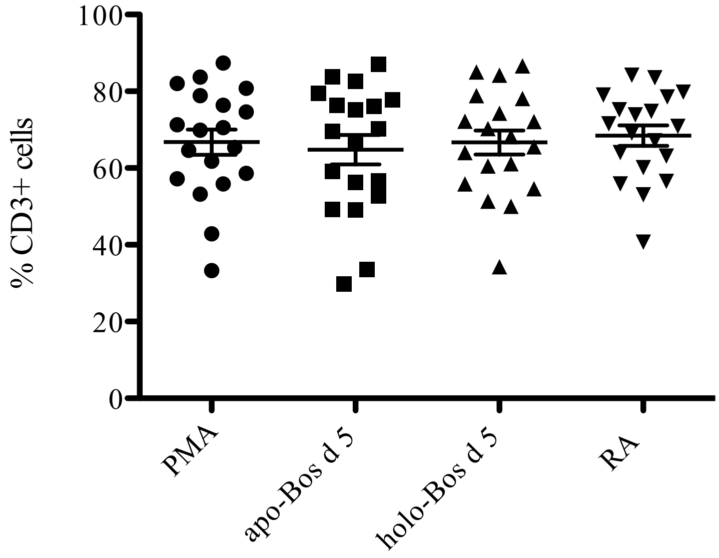

Supplementary Figure S3: TGF-ß1 levels of PBMCs stimulated during 48 hours with substances given at x-axis. Statistical analysis was performed with repeated measures ANOVA following Newman-Keuls Multiple Comparison test. * p < 0.05

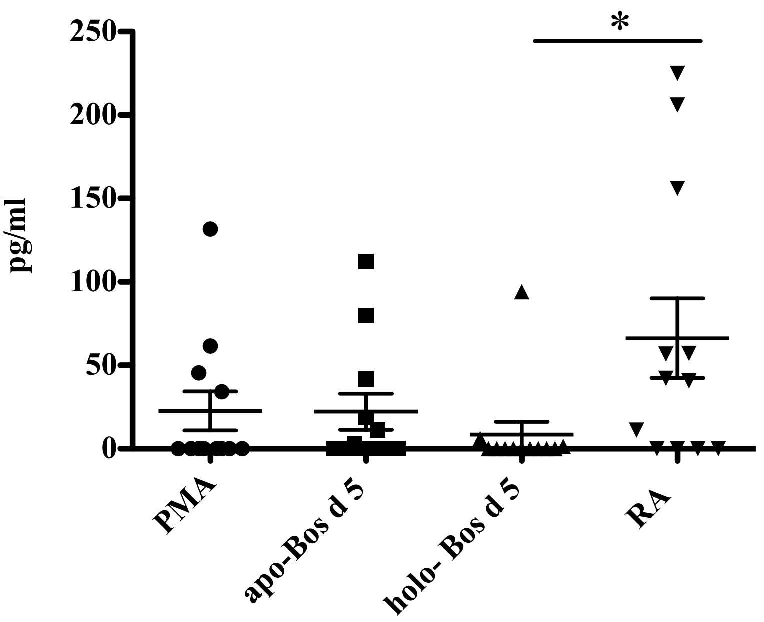

Supplementary Figure S4: Overlap of the immunodominant T-cell epitope region, the RA binding site region and predicted protease cleavage site regions. (A) Detail of the Bos d 5 amino acid sequence with the experimentally derived immunodominant T-cell epitope residues (coloured in red), the region involved in RA binding (underlined) and the predicted cathepsin S cleavage sites (coloured in blue). (B) Top 10 ranking of cathepsin S cleavage sites according to http://lightning.med.monash.edu.prosperous.

(A)

^{98}DYKKYLLFCMENSAEPEQSL^{117}

^{98}DYKKYLLFCMENSAEPEQSL^{117}

(B)

Cathepsin S cleavage site ranking:

Rank	Position	Site	Score	Family
1	7	KGLDIQ	0.73	C01.034
2	109	SAEPEQ	0.50	C01.034
3	79	AVFKID	0.47	C01.034
4	99	KKYLLF	0.45	C01.034
5	29	SLLDAQ	0.40	C01.034
6	24	AASDIS	0.39	C01.034
7	111	EPEQSL	0.37	C01.034
8	44	ELKPTP	0.36	C01.034
8	130	EALEKF	0.36	C01.034
9	137	KALKAL	0.34	C01.034
9	4	QTMKGL	0.34	C01.034
10	89	NKVLVL	0.33	C01.034

Supplementary Figure S5: **(A) Native SDS-PAGE-gel** of **α-lactalbumin** (14.2 kD, L5358, SIGMA) in native (lane 1) and pasteurized (lane 2) condition, of **Bos d 5** (18.2 kD, L0130, SIGMA) in native (lane 3) and pasteurized (lane 4) condition and of **casein** (32 kD, C7078, SIGMA) in native (lane 5) and pasteurized (lane 6) condition (Roth-Walter et al., Allergy, 2008).

All samples were applied to the same gel with one empty slot between α-lactalbumin, Bos d 5 and casein preparations (see original gel picture in **(B)**). For better visualization these empty slots between lane 2 and 3 and between lane 4 and 5 have been removed and each 2 samples of α-lactalbumin, Bos d 5 and casein have been framed with black lines. Samples were not subjected to any quantitative comparison.

(A)

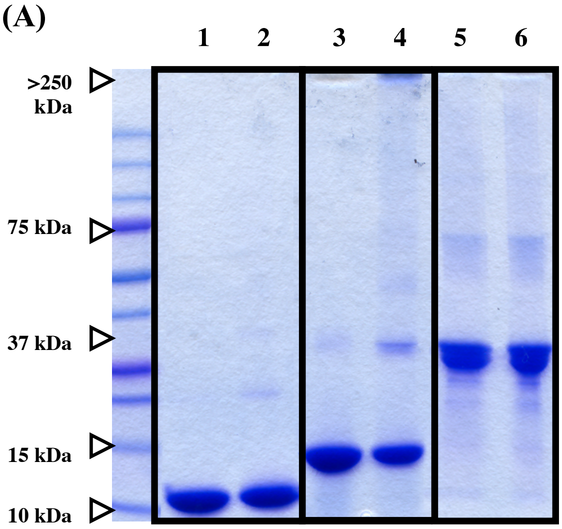

(B)

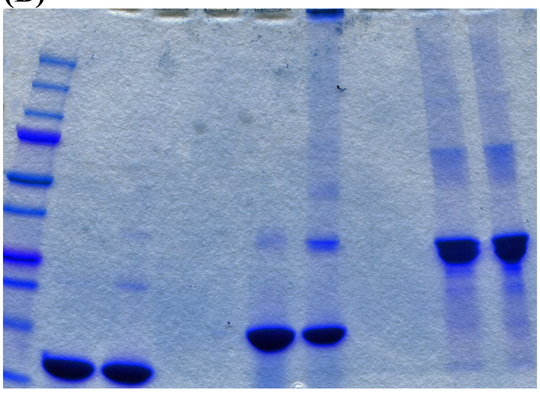

Supplementary Table S1: HLA-DRB1 alleles of five healthy donors

	Allele 1	Allele 2
Donor 1	*07	*15
Donor 2	*07	*13
Donor 3	*03	*08
Donor 4	*08	*16
Donor 5	*07	*13
